# Toughening Weak Polyampholyte Hydrogels with Weak Chain Entanglements via a Secondary Equilibrium Approach

**DOI:** 10.3390/polym15122644

**Published:** 2023-06-10

**Authors:** Tao Liu, Wenjun Chen, Kai Li, Shijun Long, Xuefeng Li, Yiwan Huang

**Affiliations:** 1 Hubei Provincial Key Laboratory of Green Materials for Light Industry, Hubei University of Technology, Wuhan 430068, China; 2 New Materials and Green Manufacturing Talent Introduction and Innovation Demonstration Base, Hubei University of Technology, Wuhan 430068, China; 3 Non-Power Nuclear Technology Collaborative Innovation Center, Hubei University of Science and Technology, Xianning 437100, China; 4 Hubei Longzhong Laboratory, Xiangyang 441000, China

**Keywords:** polyampholyte gel, topological entanglement, ionic bond, metal coordination bond, strengthening and toughening, hybrid gel

## Abstract

Polyampholyte (PA) hydrogels are randomly copolymerized from anionic and cationic monomers, showing good mechanical properties owing to the existence of numerous ionic bonds in the networks. However, relatively tough PA gels can be synthesized successfully only at high monomer concentrations (*C*_M_), where relatively strong chain entanglements exist to stabilize the primary supramolecular networks. This study aims to toughen weak PA gels with relatively weak primary topological entanglements (at relatively low *C*_M_) via a secondary equilibrium approach. According to this approach, an as-prepared PA gel is first dialyzed in a FeCl_3_ solution to reach a swelling equilibrium and then dialyzed in sufficient deionized water to remove excess free ions to achieve a new equilibrium, resulting in the modified PA gels. It is proved that the modified PA gels are eventually constructed by both ionic and metal coordination bonds, which could synergistically enhance the chain interactions and enable the network toughening. Systematic studies indicate that both *C*_M_ and FeCl_3_ concentration ($C_{{\mathrm{FeCl}}_{3}}$) influence the enhancement effectiveness of the modified PA gels, although all the gels could be dramatically enhanced. The mechanical properties of the modified PA gel could be optimized at *C*_M_ = 2.0 M and $C_{{\mathrm{FeCl}}_{3}}$ = 0.3 M, where the Young’s modulus, tensile fracture strength, and work of tension are improved by 1800%, 600%, and 820%, respectively, comparing to these of the original PA gel. By selecting a different PA gel system and diverse metal ions (i.e., Al^3+^, Mg^2+^, Ca^2+^), we further prove that the proposed approach is generally appliable. A theoretical model is used to understand the toughening mechanism. This work well extends the simple yet general approach for the toughening of weak PA gels with relatively weak chain entanglements.

## 1. Introduction

Polymeric hydrogels, consisting of three-dimensional networks, can absorb and retain a large amount of water [1,2], and show great application potential in biomedical engineering, soft electronics, information storage and encryption, and so on [3,4,5,6,7,8,9,10,11,12,13,14,15,16,17,18,19,20,21]. However, traditional hydrogels are usually brittle due to the network inhomogeneity and the lack of energy dissipation mechanism [22]. In the recent 20 years, a great progress has been made on developing strong and tough hydrogels [23,24,25,26,27,28,29,30]. As a representative tough gel, double-network (DN) hydrogels are composed of a densely cross-linked first network and a loosely cross-linked second network, providing a universal energy dissipation mechanism (i.e., sacrificial bond principle) for developing high-performance gels [22,23].

On the basis of the sacrificial bond principle, researchers have introduced dynamic physical bonds (e.g., ionic bond, hydrogen bond, metal coordination bond, and hydrophobic association) as reversible sacrificial bonds to develop tough and self-healable hydrogels, similar to that shown in soft tissues [31,32,33,34,35,36,37,38,39,40,41,42,43,44,45]. Tough and self-recoverable DN gels were fabricated by introducing Ca^2+^ ions into the first network to form metal coordination bonds, but they show relatively low load-bearing properties [46]. Instead of the DN approach, polyampholyte (PA) hydrogels have been synthesized from cationic and anionic monomers via a facile random copolymerization [47,48]. After dialysis in water, numerous dynamic ionic bonds (i.e., Coulombic attraction) could be formed between oppositely charged groups in the PA networks, enabling good mechanical properties and unique self-healing ability. It has been proved that PA gels also possess a good biocompatibility and a low cell-cytotoxicity [47]. The good mechanical properties of the gels are attributed to the self-assembled bicontinuous soft/hard multiphase network structure during the dialysis, which has been proved to be generally applicable for developing tough gels [49,50]. In our recent study, we have developed a secondary equilibrium approach to further enhance the relatively tough PA gels via the synergy of ionic and metal coordination bonds [45]. However, a tough PA hydrogel can be synthesized near the charge balance point successfully only at a relatively high monomer concentration (*C*_M_); otherwise, a relatively low *C*_M_ usually results in a weak PA gel with limited chain entanglements because of the unstable primary topological architecture [47,48]. This phenomenon indicates that the effective chain entanglement is needed for achieving stable supramolecular networks, which could further promote the formation and enhancement of numerous ionic bonds, resulting in tough PA gels.

**Figure 1:**
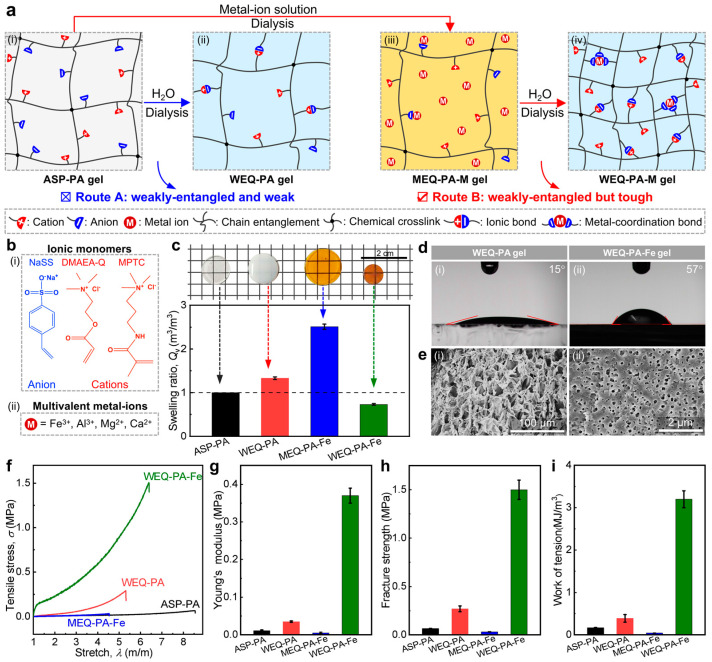
Design, preparation, structures, and mechanical proof. (**a**) Design and fabrication of WEQ-PA gels (**Route A**) and WEQ-PA-M (**Route B**) gels. **Route A** represents the dialysis of ASP-PA gel only in water. **Route B** represents the dialysis of ASP-PA gel in metal ion solution and water in sequence through a two-step process. “ASP”, “MEQ”, and “WEQ” represent as-prepared, metal-ions-equilibrated, and water-equilibrated states, respectively. For simplicity, the corresponding counter ions are not shown here. (**b**) Chemical structures of ionic monomers and multivalent metal ions used in this study. (**c**) Macroscopic images and volume swelling ratio (*Q*_v_) of four representative samples ($C_{M}$ = 1.7 M, $C_{{\mathrm{FeCl}}_{3}}$ = 0.7 M). Here the original PA gel is P(NaSS-*co*-DMAEA-Q) gel. (**d**,**e**) Contact angle (**d**) and SEM (**e**) images of the corresponding WEQ-PA gel and WEQ-PA-Fe gel. (**f**–**i**) Tensile stress–stretch ratio curves (**f**) and detailed tensile data (**g**–**i**) of the four representative samples. Values in (**c**,**g**–**i**) are expressed as mean value ± SD (*n* ≥ 3).

**Figure 2:**
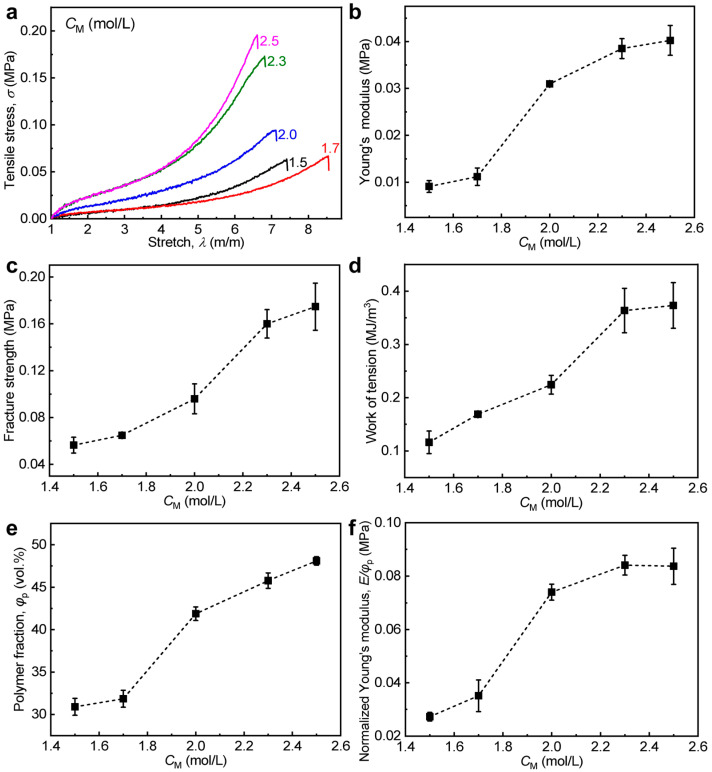
Tensile behavior of ASP-PA gels with different *C*_M_. Here the original PA gel is P(NaSS-*co*-DMAEA-Q) gel. (**a**) Tensile stress–stretch ratio curves. (**b**–**d**) Detailed Young’s modulus (**b**), tensile fracture strength (**c**), and work of tension (**d**) versus *C*_M_. (**e**) Volume fraction of polymer (*φ*_p_) of the gels versus *C*_M_. (**f**) Normalized Young’s modulus (*E*/*φ*_p_) versus *C*_M_. Values in (**b**–**f**) are expressed as mean value ± SD (*n* ≥ 3).

In an extension of our recent work on the mechanical enhancements of relatively tough PA hydrogels with a relatively high *C*_M_ [45], this study aims to toughen weak PA gels with relatively weak primary chain entanglements via a secondary equilibrium approach (Figure 1a). The chain entanglements are tuned by varying the monomer concentration in the pre-gel solutions (Figure 2). In this approach, an as-prepared PA gel is first dialyzed in a multivalent metal ion solution to reach a swelling equilibrium (Step-I) and then moved to deionized water to dialyze out excess free ions sufficiently to achieve a new equilibrium (Step-II), enabling the toughening of the weak PA gels. According to the approach, we first chose a model PA gel with a relatively low *C*_M_ (1.7 M), P(NaSS-*co*-DMAEA-Q) gel [randomly polymerized from sodium *p*-styrenesulfonate (NaSS), and methyl chloride quarternized *N*,*N*-dimethylamino ethylacrylate (DMAEA-Q)], and a model metal ion solution, i.e., 0.7 M FeCl_3_ solution. The result demonstrates that the PA gel with relatively low chain entanglements could be effectively toughened through our approach. Further systematic studies were also carried out by varying *C*_M_ (1.5–2.5 M) and FeCl_3_ concentration ($C_{{\mathrm{FeCl}}_{3}}$ = 0–2.0 M) to understand their effects on the mechanics of the gels. The results show that the enhancement effectiveness is different although all the PA gels with different *C*_M_ could be mechanically enhanced. Meanwhile, $C_{{\mathrm{FeCl}}_{3}}$ could also result in different mechanical enhancements by influencing the quantity and quality of the introduced metal coordination bonds in the PA networks. The optimal mechanical improvement of the modified PA gels could be achieved at *C*_M_ = 2.0 M and $C_{{\mathrm{FeCl}}_{3}}$ = 0.3 M, where Young’s modulus, tensile fracture strength, and work of tension were considerably improved by 1800%, 600%, and 820%, respectively, in comparison to those of the original PA gel. We further selected another PA gel system, P(NaSS-*co*-MPTC) [synthesized like the former gel system, where the monomer DMAEA-Q was replaced by 3-(methacryloylamino)propyl-trimethylammonium chloride (MPTC)], and various multivalent metal ions (i.e., Al^3+^, Mg^2+^, Ca^2+^) to understand the generality of our proposed approach. These experiments confirmed that the approach is relatively general for toughening PA gels with relatively low *C*_M_. A theoretical model was also adopted to disclose the toughening mechanism of the gels. This work well extends the simple yet general way for the toughening of weak PA gels with relatively weak chain entanglements.

## 2. Materials and Methods

### 2.1. Materials

Sodium *p*-styrenesulfonate (NaSS, 90 wt%; anionic monomer) was purchased from Shanghai Macklin Biochemical Co., Ltd. (Shanghai, China). Dimethylaminoethylacrylate quaternized ammonium (DMAEA-Q, 80 wt%; cationic monomer) and 3-(methacryloylamino)propyl-trimethylammonium chloride (MPTC, 50 wt%; cationic monomer) were purchased from J&K Chemical Ltd. *N*,*N*′-methylene-bis-acrylamide (MBAA; chemical crosslinker), *α*-ketoglutaric acid (*α*-keto; ultraviolet photo-initiator), and metal ion salts were purchased from Sinopharm Chemical Reagent Co., Ltd. (Shanghai, China). Chemical structures of the ionic monomers are presented in Figure 1b(i). All reagents were of analytical grade and were used as received. Deionized (DI) water (18.3 MΩ) was used in all the experiments.

### 2.2. Preparation of Polyampholyte (PA) Hydrogels

P(NaSS-*co*-DMAEA-Q) hydrogel was prepared by a free radical polymerization. Typically, a pre-gel solution containing anionic monomer (NaSS), cationic monomer (DMAEA-Q), cross-linker (MBAA), and photoinitiator (*α*-keto) was prepared according to previous studies [47,51,52]. The total ionic monomer concentration (*C*_M_) widely ranged from 1.5 to 2.5 M, and the molar fraction of anionic monomer (*f*_a_) was fixed as 0.51. The molar fractions of both crosslinker and initiator were 0.10 mol%, relative to *C*_M_. The pre-gel solution was injected into a reaction cell consisting of a pair of glass plates as walls and a silicone spacer (geometry: 10 cm width × 10 cm length × 1 mm thickness). The reaction cell was then irradiated by an ultraviolet lamp (365 nm, 4 W cm^−2^) for polymerization for 10 h at ambient temperature, to form as-prepared PA (ASP-PA) hydrogels. After that, the ASP-PA gels were immersed in a large amount of DI water for around 1 week to remove residual chemicals until a new equilibrium was achieved. To ensure sufficient dialysis, DI water was refreshed every 12 h. During this process, mobile counter ions were dialyzed gradually, and dynamic ionic bonds were probably formed between the oppositely charged groups on the polymer chains through Coulombic electrostatic attraction. For simplification, the water-equilibrated PA gels were denoted as WEQ-PA gels. Another cationic monomer (MPTC) was also used instead of DMAEA-Q to prepare P(NaSS-*co*-MPTC) hydrogel by the same procedure. For identification, generally the mentioned PA gel was P(NaSS-*co*-DMAEA-Q) gel in this work; otherwise, the gel system detail would be given.

### 2.3. Preparation of PA-M Hydrogels

Fe^3+^ ions filled PA (PA-Fe) hydrogels were prepared by a facile secondary equilibrium approach. Typically, an ASP-PA gel was first soaked in a 200 mL FeCl_3_ solution at ambient temperature until a new equilibrium was achieved, where the solution did not change. The FeCl_3_ concentration ($C_{{\mathrm{FeCl}}_{3}}$) of the soaking solutions ranged from 0 to 2.0 M. After around 3 days, ASP-PA-Fe gels were obtained, followed by re-dialysis in adequate DI water for at least 1 week. In the re-dialysis step, excessive Fe^3+^ ions and the corresponding counter ions were dialyzed out completely to reach a secondary equilibrium, resulting in tough WEQ-PA-Fe gels. To guarantee sufficient dialysis, DI water was refreshed every 12 h. To verify the proposed approach, both PA hydrogel systems (i.e., P(NaSS-*co*-DMAEA-Q) and P(NaSS-*co*-MPTC) gels) were used to prepare ASP-PA-Fe gels. Meanwhile, some other metal ion solutions (i.e., AlCl_3_, MgCl_2_, CaCl_2_, and NaCl solutions, *C*_M_ = 0.3 M) were also chosen as the soaking solutions to prepare ASP- and WEQ-PA-M hydrogels by the same procedure, where M represents the multi- or mono-valent metal ions mentioned above. For identification, the hydrogel samples were denoted as PA-*C*_M_-M-*C*_metal_, where *C*_metal_ is the concentration of metal ion solutions.

### 2.4. Volume Swelling Ratio of Hydrogels

The original hydrogel samples were immersed in metal ion solutions to achieve the equilibrium state, and then immersed in DI water to achieve the secondary re-equilibrium state. The original volume of the as-prepared cylindrical sample is *V*_0_ [geometry: original diameter *d*_0_ (10.7 mm) and original thickness *t*_0_ (≈1 mm)], and the volume at a different state is *V* (diameter *d* and thickness *t*). Volume swelling ratio (*Q*_v_) of the samples is defined as [45,52]:

(1) $$Q_{v}=\frac{V}{V_{0}}=\frac{d^{2}\cdot t}{{d_{0}}^{2}\cdot t_{0}}$$

Each sample was measured for at least three times, the average value was calculated, and the standard deviation was obtained as error bar.

### 2.5. Scanning Electron Microscopy (SEM)

Cross-sectional morphologies of the hydrogel samples were observed by a Hitachi SU8010 field emission SEM. In order to guarantee the effective observations of the microstructures, the samples were frozen and fractured in liquid nitrogen and then freeze-dried for 24 h [37,39,45]. Afterwards, the fractured surfaces of the samples were gold-coated in a JUC-500 Magnetron Sputtering Device (JEOL, Tokyo, Japan), and then observed with an accelerating voltage of 3 kV.

### 2.6. Contact Angle Measurements

Static contact angles (*θ*_con_) of the samples were measured using an optical contact angle measuring system (JC-2000D, Beijing Zhongyi Kexin Technology Co., Ltd., Beijing, China). DI water droplets were deposited on a flat and horizontal sample surface. The droplet size was adjusted by means of a Microsyringe coupled to a programmable pump with slow (5 mL min^−1^) injection speed to keep the 3-phase contact in equilibrium. Images of the droplets were digitized and analyzed with a software to evaluate the contact angles.

### 2.7. Tensile Tests

Uniaxial tensile tests of the samples were performed by using a universal testing machine (E43.104, MTS, Shanghai, China) with a 200 N load cell (standard: JIS-K6251-7). Before the tests, the samples were cut into a dumbbell shape (gauge length *l* = 12 mm, width *w* = 2 mm, and thickness *t* = 1–2 mm) (Figure S2). The tests were performed at ambient temperature with a stretch velocity of 100 mm min^−1^. To prevent hydrogel dehydration, a humidifier was used to directly spray to the samples during the tests. Young’s modulus, *E*, was calculated from the initial slope of the stress–strain curves at the tensile stain within 10%. Work of tension of the samples during the tests (*W*_b_) was calculated by integrating the area under the stress–strain curves as follows:

(2) $$W_{b}=\int_{0}^{\varepsilon_{b}}\sigma d\varepsilon$$

where *σ* and *ε* were the stress and strain, respectively, and *ε*_b_ was the strain at breaking of the samples [45].

## 3. Results and Discussion

### 3.1. Design, Preparation, and Characterizations

The design and preparation process of metal ion filled PA hydrogels with relatively weak primary chain entanglements are shown in Figure 1a. For simplification, the as-prepared PA gel, the water-equilibrated PA gel, the PA gel immersed in a metal ion solution, and the water-equilibrated PA gel after immersion in a metal ion solution are denoted as ASP-PA gel, WEQ-PA gel, MEQ-PA-M gel, and WEQ-PA-M gel, respectively, where M represents Fe^3+^, Al^3+^, Mg^2+^, and Ca^2+^ ions (Figure 1b(ii)). Two routes are presented to achieve the water-equilibrated gels in Figure 1a. In Route A (from ASP-PA gel to WEQ-PA gel), the Coulomb repulsion of the network tends to prevail over the limited Coulomb attraction of oppositely charged groups due to the relatively weak primary chain entanglements, leading to the swelling of the network and the mechanical weakness. Route B is our proposed approach that contains two steps in the soaking process. In Step-I from ASP-PA gel to MEQ-PA-M gel, metal ions and their counter ions diffuse into the network due to the osmotic difference between inside and outside of the network, resulting in the de-association of originally formed limited ionic bonds and the swelling of the network. In Step-II from MEQ-PA-M gel to WEQ-PA-M gel, the PA network with relatively weak primary chain entanglements is possibly optimized and constructed by both ionic and metal coordination bonds after excess metal ions and their counter ions are dialyzed out, enabling the mechanical enhancements of the hydrogels.

The mechanical contribution of ASP-PA gels is mainly related to the polymer chain density, the chain entanglements, and the Coulomb electrostatic attraction of oppositely charged groups (i.e., ionic bonds) in the network. Due to the shielding effect of numerous counter ions, ionic bonds in the network should be very limited and comparable in the weak ASP-PA gels with different monomer concentrations (*C*_M_) [47,48]. In addition, the effect factor of polymer chain density could be also normalized to simplify the mechanical contribution on the basis of classical rubber elasticity theory [53]. In this case, it seems possible to distinguish the difference of chain entanglements by varying *C*_M_ in the pre-gel solutions in the ASP-PA gels. As shown in Figure 2a–d, the tensile behavior of ASP-PA gels with different *C*_M_ (1.5–2.5 M) is presented. It is clear that the tensile properties of the gels increase gradually at *C*_M_ = 1.5–2.3 M and is relatively stable after *C*_M_ ≥ 2.3 M. The volume polymer fraction (*φ*_p_) increases from 30.9 vol.% to 48.1 vol.% with increasing *C*_M_ (1.5–2.5 M), which played a role in the mechanical enhancements (Figure 2e), while water content ($\omega_{w}$) decreases gradually (Figure S3). To exclude the effect of *φ*_p_, we further normalized the Young’s modulus of the ASP-PA gels by *φ*_p_, as shown in Figure 2f. Here *φ*_p_ was calculated as follows [54]:

(3) $$\varphi_{p}^{-1}=1+\left(\frac{\omega_{w}}{1\;-\omega_{w}}\right)\frac{\rho_{p}}{\rho_{w}}$$

where $\omega_{w}$, *ρ*_p_, and *ρ*_w_ are the water content of the hydrogel, the density of polymer (≈1.08 g cm^−3^), and the density of water (≈0.998 g cm^−3^), respectively. After the normalization, the mechanical difference should be mainly due to the difference of chain entanglements. Clearly, the chain entanglements became stronger gradually with increasing *C*_M_ (1.5–2.3 M), enhancing the mechanical properties; they became comparable after *C*_M_ ≥ 2.3 M, resulting in the relatively stable mechanical properties.

Based on the above result, to verify our proposed approach, we first chose a P(NaSS-*co*-DMAEA-Q) gel (*C*_M_ = 1.7 M) as a model PA gel with relatively weak primary chain entanglements and 0.7 M FeCl_3_ solution as a model metal ion solution for dialysis to prepare a WEQ-PA-Fe gel. After immersing ASP-PA gel in water directly, WEQ-PA gel showed a swelling behavior (volume swelling ratio *Q*_v_ = 1.33 m^3^ m^−3^) and a turbid appearance, indicating the occurrence of clear phase separation in the network (Figure 1c). In comparison, when ASP-PA gel was immersed in the FeCl_3_ solution and water in sequence, the resulting MEQ-PA-Fe gel showed a relatively large *Q*_v_ (2.51 m^3^ m^−3^), but WEQ-PA-Fe gel showed a clear deswelling behavior (*Q*_v_ = 0.55 m^3^ m^−3^). The swelling of WEQ-PA gel is mainly due to the relatively weak primary chain entanglements, making the Coulomb repulsion dominate the behavior in the network. Through our approach, the yellow-brown color of WEQ-PA-Fe gel indicates the successful introduction of metal coordination bonds (Figure 1c), which has been further evidenced by the FTIR and XRF data (Figure S4 and Table S1). After the introduction of Fe^3+^ ions, almost all characteristic peaks of −SO_3_^–^ groups in the FTIR spectrum redshift and become stronger and broader in WEQ-PA-Fe gel, suggesting the formation of metal coordination (–SO_3_^−^•••Fe^3+^) bonds (Figure S4). Meanwhile, the characteristic peaks of −(CH_3_)_3_N^+^ groups also become stronger and broader in WEQ-PA-Fe gel, indicating the strengthening of the ionic bonds between the oppositely charged groups. The XRF result further confirms the existence of the ferric element in WEQ-PA-Fe gel (Table S1). The clear deswelling behavior of WEQ-PA-Fe gel should be mainly attributed to the synergy of the formed ionic and metal–coordination bonds.

Static contact angle (*θ*_con_) tests show that such possibly formed metal coordination bonds could clearly affect the hydrophilicity of the hydrogel surface (Figure 1d). The hydrophilicity of PA gels should be mainly related to the water content and unpaired charged groups, which could influence the network polarity [52,55]. WEQ-PA gel shows a relatively hydrophilic surface (*θ*_con_ ≈ 15°) due to its polar property. In comparison, WEQ-PA-Fe gel possesses a relatively high *θ*_con_ (≈57°), indicating a relatively poorer hydrophilicity probably mainly because of the formed ionic and metal coordination bonds. The formation of these dynamic bonds also influenced the microstructure of the PA gel, as indicated by SEM images (Figure 1e). The freeze-dried WEQ-PA gel exhibits a loose structure, but WEQ-PA-Fe gel shows a relatively dense structure. This structural difference might be also induced by the synergy of the formed ionic and metal coordination bonds, which should be beneficial to the mechanics of the gels.

We then compare the tensile behavior of the hydrogel samples (Figure 1f–i). Due to the lack of energy dissipation mechanism, both ASP-PA gel and MEQ-PA-Fe gel show particularly weak tensile performance. After dialysis in water, WEQ-PA gel was just mechanically enhanced mildly compared with ASP-PA gel but still relatively weak, indicating that relatively strong primary chain entanglements are necessary for the formation of tough PA gels with numerous ionic bonds, agreeing with previous studies [47,48]. Based on our proposed approach, WEQ-PA-Fe gel shows significant mechanical enhancements: 0.37 MPa of Young’s modulus (*E*), 1.5 MPa of tensile fracture strength (*σ*_b_), and 3.2 MJ m^−3^ of work of tension at break (*W*_b_), which are 10.6, 5.6, and 8.2 times these of WEQ-PA gel, respectively (Figure 1g–i). The mechanical data clearly prove that PA gels with relatively weak primary chain entanglements could be effectively enhanced by the synergy of ionic and metal coordination bonds, supporting our proposed approach very well.

To verify the synergistic effect of the two kinds of dynamic bonds on the mechanical properties of PA gels with relatively weak primary chain entanglements, we prepared a control gel by using a neutral monomer [i.e., acrylamide (AAm)] instead of the cationic monomer (i.e., DMAEA-Q) in P(NaSS-*co*-DMAEA-Q) gel (Figure S5 and Table S2). The stoichiometric ratio is the same as the PA gels, and the sample is simply named as P(NaSS-*co*-AAm) gel (shortly as PNA gel). WEQ-PNA-Fe gel with *C*_M_ = 2.0 M and $C_{{\mathrm{FeCl}}_{3}}$ = 0.3 M was further prepared via our proposed approach, where only metal coordination (–SO_3_^−^•••Fe^3+^) bonds but without ionic bonds could be formed. The tensile result demonstrates that the mechanical properties of WEQ-PA-Fe gel highly surpass these of WEQ-PNA-Fe gel, indicating that both ionic and metal coordination bonds synergistically enhanced the PA gels.

### 3.2. Effect of Monomer Concentration in Pre-Gel Solutions

As aforementioned, *C*_M_ could influence the primary chain entanglements and polymer chain density of the as-prepared PA (ASP-PA) gels, which accordingly resulted in different mechanical properties. We hypothesize that the mechanical enhancements of WEQ-PA-Fe gels could be also affected by *C*_M_. In this section, we systematically study the effect of *C*_M_ on the tensile behaviors of the gels.

**Figure 3:**
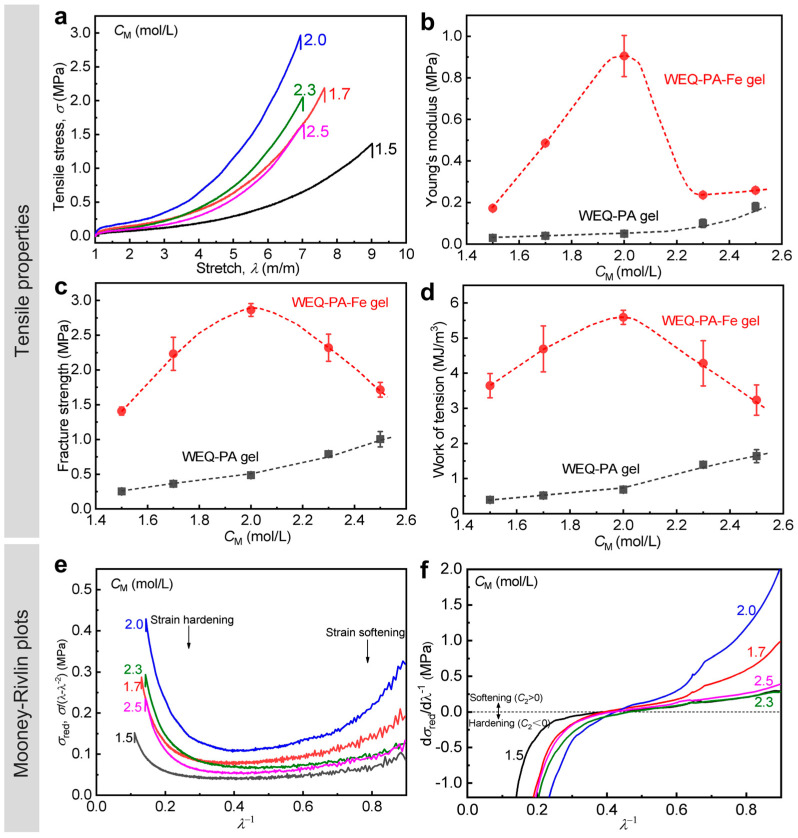
Tensile behavior of WEQ-PA-Fe hydrogels with different *C*_M_. (**a**) Tensile stress–stretch ratio curves. (**b**–**d**) Detailed Young’s modulus (**b**), tensile fracture strength (**c**), and work of tension (**d**) versus *C*_M_. (**e**) Reduced stress ($\sigma_{\mathrm{red}}$) versus *λ*^−1^ curves and (**f**) the corresponding derivation curves based on the Mooney–Rivlin model. Values in (**b**–**d**) are expressed as mean value ± SD (*n* ≥ 3). The curves shown in (**f**) are smoothed to reduce the noise but not to influence the trend of the data. Dashed lines are manually drawn as visual guides. $C_{{\mathrm{FeCl}}_{3}}$ = 0.3 M.

We chose 0.3 M FeCl_3_ solution as a model metal ion solution and varied *C*_M_ at 1.5–2.5 M to fabricate a series of WEQ-PA-Fe gels. The detailed tensile properties of the gels are presented in Figure 3a–d, Table 1 and Table S3. The data show that all the WEQ-PA-Fe gels with different *C*_M_ were mechanically enhanced clearly compared to WEQ-PA gels. The result demonstrates that the secondary equilibrium approach (that was firstly proposed previously [45]) is successfully extended for the toughening of PA gels with relatively weak primary chain entanglements (1.5 M ≤ *C*_M_ ≤ 2.0 M). Importantly, these PA gels with relatively weak chain entanglements could be enhanced more significantly, compared with these gels with relatively high chain entanglements (*C*_M_ > 2.0 M) (that was also reported previously) [45]. When *C*_M_ is even as low as 1.5 M, WEQ-PA-Fe gel exhibits 0.17 MPa of Young’s modulus (*E*), 1.4 MPa of tensile fracture strength (*σ*_b_), and 3.6 MJ m^−3^ of work of tension at break (*W*_b_), which are 28.3, 175, and 900 times these of the corresponding neat WEQ-PA gel, respectively. WEQ-PA-Fe gel possesses the highest mechanical properties at *C*_M_ = 2.0 M: *E* = 0.90 MPa, $\sigma_{b}$ = 2.9 MPa, and $W_{b}$ = 5.6 MJ m^−3^, which are 18, 6.0, and 8.2 times these of the corresponding neat WEQ-PA gel, respectively. The significant mechanical improvements should be attributed to the high synergy efficiency of the formed ionic and metal coordination bonds, which have been confirmed by the FTIR result (Figure S6).

Based on the tensile curves of the gels (Figure 3a), the strain softening and hardening behaviors are also understood by a theoretical Mooney–Rivlin model [54,56,57,58]:

(4) $$\sigma_{\mathrm{red}}=\frac{\sigma }{\lambda -\lambda^{-2}}=2C_{1}+2C_{2}\frac{1}{\lambda }$$

where $\sigma_{\mathrm{red}}$ is the reduced stress, *λ* is the stretch ratio, and C_1_ and C_2_ are the material constants. 2C_1_ is equal to the shear modulus, *G* (≈*E*/3), and C_2_ is related to the strain softening (C_2_ > 0) and hardening (C_2_ < 0) beyond Gaussian elasticity region. A material experiences a purely elastic stretching region when C_2_ = 0. We plot $\sigma_{\mathrm{red}}$ versus *λ*^−1^ curves and their derivation curves (corresponding to the slope changes) of WEQ-PA-Fe gels with different *C*_M_ (Figure 3e,f). It can be observed that all the WEQ-PA-Fe gels show clear strain softening and hardening behaviors. In neat WEQ-PA gels with relatively high *C*_M_, relatively weak ionic bonds serve as sacrificial bonds to contribute to the softening behavior, while relatively strong ionic bonds and covalent bonds are responsible for the hardening behavior [47,48]. In our WEQ-PA-Fe gels, relatively weak ionic and metal coordination bonds are considered to contribute to the softening behavior, and relatively strong ionic and metal coordination bonds as well as covalent bonds are related to the hardening behavior. The Mooney–Rivlin plots tell that both relative weak and strong bonds were enhanced dramatically owing to the introduction of metal coordination bonds. The more prominently enhanced hardening behavior further indicates that more strong bonds were formed in the gels. In addition, the two behaviors are the strongest for WEQ-PA-Fe gels with *C*_M_ = 2.0 M, indicating the highest synergy efficiency in mechanical enhancements. The above result further evidenced that our proposed approach is effective for enhancing the weak PA gels with relatively weak primary chain entanglements.

### 3.3. Effect of Metal ion Concentration of Dialysis Solution

Metal ion concentration of dialysis solution could probably determine the metal ion loading in the gels, which might affect the quantity and quality of the formed metal coordination bonds, accordingly resulting in different mechanical improvements. Therefore, we next investigate the effect of the concentration of FeCl_3_ solution ($C_{{\mathrm{FeCl}}_{3}}$) on the mechanics of WEQ-PA-Fe gels with relatively weak primary chain entanglements.

**Figure 4:**
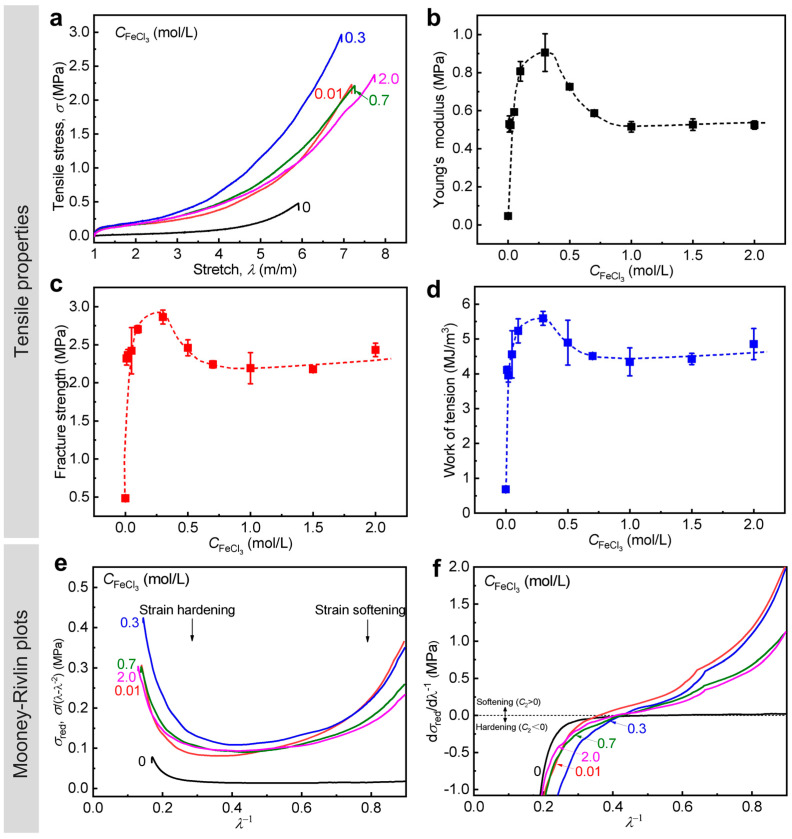
Tensile behavior of WEQ-PA-Fe hydrogels with different $C_{{\mathrm{FeCl}}_{3}}$. (**a**) Tensile stress–stretch ratio curves. (**b**–**d**) Detailed Young’s modulus (**b**), tensile fracture strength (**c**), and work of tension (**d**) versus $C_{{\mathrm{FeCl}}_{3}}$. (**e**) Reduced stress ($\sigma_{\mathrm{red}}$ ) versus *λ*^−1^ curves and (**f**) the corresponding derivation curves based on the Mooney–Rivlin model. Values in (**b**–**d**) are expressed as mean value ± SD (*n* ≥ 3). The curves shown in (**f**) are smoothed to reduce the noise but not to influence the trend of the data. Dashed lines are manually drawn as visual guides. *C*_M_ = 2.0 M.

We chose the PA gel with *C*_M_ = 2.0 M as a representative gel and varied $C_{{\mathrm{FeCl}}_{3}}$ at 0–2.0 M to fabricate a series of WEQ-PA-Fe gels. Their tensile properties are given in Figure 4a–d and Table 1. The tensile data show that all the WEQ-PA-Fe gels could be enhanced significantly. The different enhancements also confirmed the clear influence of $C_{{\mathrm{FeCl}}_{3}}$ on the mechanical behavior of the gels. In detail, the tensile properties increase continuously at 0 < $C_{{\mathrm{FeCl}}_{3}}$ ≤ 0.1 M, then become comparable at 0.1 < $C_{{\mathrm{FeCl}}_{3}}$ ≤ 0.3 M, and decrease mildly but are still much higher than the corresponding neat gel until $C_{{\mathrm{FeCl}}_{3}}$ ≥ 0.3 M. The tendency is also similar to our recent data on the toughening of PA gels with relatively strong primary chain entanglements (*C*_M_ = 2.3 M) [45]. The result suggests that the synergistic effect of ionic and metal coordination bonds is effective in a wide range of $C_{{\mathrm{FeCl}}_{3}}$ and could be maximized at 0.1 < $C_{{\mathrm{FeCl}}_{3}}$ ≤ 0.3 M. The FTIR result indicates that the chemical structures of the gels were influenced by $C_{{\mathrm{FeCl}}_{3}}$ by affecting the quantity and quality of the formed ionic and metal coordination bonds (Figure S7). The XRF result further shows that the content of Fe element (*ω*_Fe_) and the atomic ratio of Fe:S ($r_{\mathrm{Fe}/S}$) increase gradually with increasing $C_{{\mathrm{FeCl}}_{3}}$ (0–0.3 M), indicating the gradually increased quantity of the formed metal coordination bonds; they become comparable after $C_{{\mathrm{FeCl}}_{3}}$ ≥ 0.5 M (Table S1). The tensile data are in good agreements with the FTIR and XRF data. On the basis of the tensile curves (Figure 4a), the strain softening and hardening behaviors of the gels with different $C_{{\mathrm{FeCl}}_{3}}$ were also studied by the Mooney–Rivlin model, and the corresponding plots are given in Figure 4e,f. Both the two kinds of behaviors became strong after the introduction of metal coordination bonds, and the influence on the hardening behaviors is more significant. The result also indicates that both relative weak and strong bonds were enhanced, and more strong bonds were formed in the gels. With increasing $C_{{\mathrm{FeCl}}_{3}}$, the change tendency of the strength of the two behaviors is similar to that shown in tensile properties. Interestingly, WEQ-PA-Fe gel could achieve relatively high mechanical properties even at $C_{{\mathrm{FeCl}}_{3}}$ = 0.01 M: *E* = 0.53 MPa, $\sigma_{b}$ = 2.3 MPa, and $W_{b}$ = 4.1 MJ m^−3^, which are 12, 4.8, and 6.0 times these of the corresponding neat gel, respectively (Figure 4b–d).

**Figure 5:**
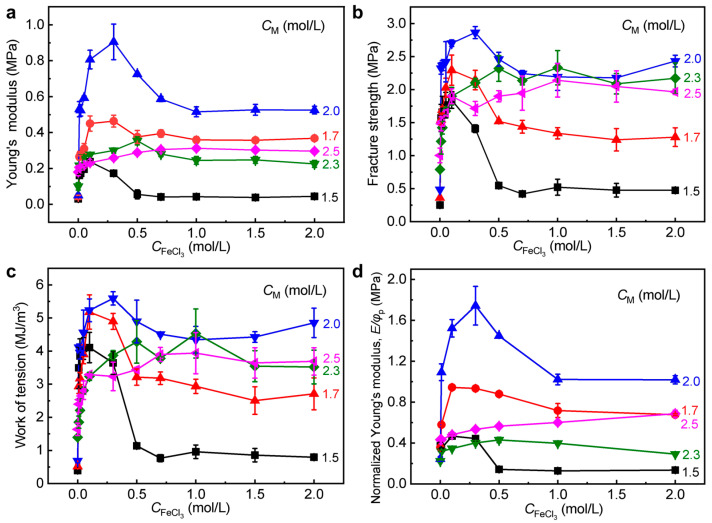
Tensile properties of WEQ-PA-Fe hydrogels with different *C*_M_ as a function of $C_{{\mathrm{FeCl}}_{3}}$. Here the original PA gel is P(NaSS-*co*-DMAEA-Q) gel. (**a**) Young’s modulus, (**b**) tensile fracture strength, and (**c**) work of tension versus $C_{{\mathrm{FeCl}}_{3}}$. (**d**) Normalized Young’s modulus (*E*/*φ*_p_) versus $C_{{\mathrm{FeCl}}_{3}}$. All values are expressed as mean value ± SD (*n* ≥ 3).

The tensile properties of WEQ-PA-Fe gels with different *C*_M_ as a function of $C_{{\mathrm{FeCl}}_{3}}$ are further presented in Figure 5a–c. It is clearly seen that with increasing $C_{{\mathrm{FeCl}}_{3}}$ the gels with different *C*_M_ show the similar trend described above for WEQ-PA-Fe gels with *C*_M_ = 2.0 M (Figure 4a–d). It is also seen that the mechanical enhancements of the gels become more prominent with increasing *C*_M_ (1.5–2.0 M), then become slightly weaker after *C*_M_ > 2.0 M. It is probably because the synergistic enhancements of the gels could be optimized at *C*_M_ = 2.0 M via the formed ionic and metal coordination bonds. The normalized *E* (i.e., *E*/*φ*_p_) data further confirmed the critical role of the synergy of the two kinds of dynamic bonds in mechanical enhancements (Figure 5d). All these results confirmed the effectiveness of our proposed approach for the mechanical improvements of the PA gels with relatively weak primary chain entanglements.

### 3.4. Generality of the Proposed Approach

**Figure 6:**
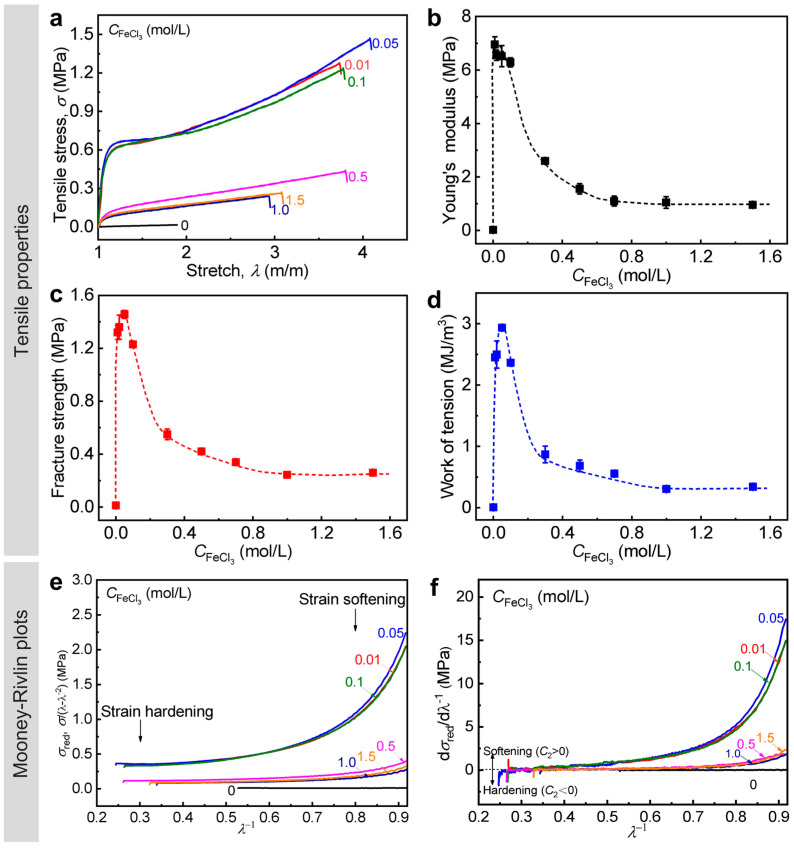
Tensile behavior of WEQ-PA-Fe hydrogels with different $C_{{\mathrm{FeCl}}_{3}}$. Here the original PA gel is P(NaSS-*co*-MPTC) gel. (**a**) Tensile stress–stretch ratio curves. (**b**–**d**) Detailed Young’s modulus (**b**), tensile fracture strength (**c**), and work of tension (**d**) versus $C_{{\mathrm{FeCl}}_{3}}$. (**e**) Reduced stress ($\sigma_{\mathrm{red}}$ ) versus *λ*^−1^ curves and (**f**) the corresponding derivation curves based on the Mooney–Rivlin model. Values in (**b**–**d**) are expressed as mean value ± SD (*n* ≥ 3). The curves shown in (**f**) are smoothed to reduce the noise but not to influence the trend of the data. *C*_M_ = 1.5 M.

To study the generality of our proposed approach, we selected another kind of PA hydrogel [i.e., P(NaSS-*co*-MPTC) gel] with a relatively low *C*_M_ (1.5 M) to prepare WEQ-PA-Fe gels with different $C_{{\mathrm{FeCl}}_{3}}$. Compared to the neat P(NaSS-*co*-DMAEA-Q) gel with the same *C*_M_, the primary neat P(NaSS-*co*-MPTC) gel possesses higher tensile properties, although it is still very weak (Figure 4 and Figure 6). In this section, the two kinds of initial PA gels are denoted as PA(DMAEA-Q) and PA(MPTC) gels for simplicity. WEQ-PA(MPTC)-Fe gels were prepared by the same process and $C_{{\mathrm{FeCl}}_{3}}$ was 0–1.5 M. Tensile properties of the gels are presented in Figure 6a–d and Table 2. Similarly, all WEQ-PA(MPTC)-Fe gels exhibit clear mechanical enhancements in comparison to the neat WEQ-PA(MPTC) gel. In detail, the tensile data of the gels increase to peak values with increasing $C_{{\mathrm{FeCl}}_{3}}$ (0–0.05 M), and then decrease to plateau values with further increasing $C_{{\mathrm{FeCl}}_{3}}$ until $C_{{\mathrm{FeCl}}_{3}}$ = 1.0 M. When $C_{{\mathrm{FeCl}}_{3}}$ = 0.05 M, WEQ-PA(MPTC)-Fe gel exhibits the highest tensile performance: *E* = 6.5 MPa, $\sigma_{b}$ = 1.5 MPa, and $W_{b}$ = 2.9 MJ m^−3^, which are 310, 125, and 414 times these of the corresponding neat gel, respectively. Based on the tensile curves (Figure 6a), the strain softening and hardening behaviors of the gels were also studied by the Mooney–Rivlin model (Figure 6e,f). Compared to the neat gel, the softening behaviors of WEQ-PA(MPTC)-Fe gels were enhanced significantly, while their hardening behaviors showed negligible enhancements. The result indicates that, through our proposed approach, the additionally formed relatively weak ionic and metal coordination bonds mainly contribute to the mechanical enhancements. With increasing $C_{{\mathrm{FeCl}}_{3}}$, the change tendency of the strength of the softening behaviors is also similar to that shown in tensile properties. It is also worth noting that WEQ-PA(MPTC)-Fe gel could also achieve relatively high mechanical properties at $C_{{\mathrm{FeCl}}_{3}}$ as low as 0.01 M: *E* = 7.0 MPa, $\sigma_{b}$ = 1.3 MPa, and $W_{b}$ = 2.5 MJ m^−3^, which are 333, 108, and 357 times these of the corresponding neat gel, respectively (Figure 6b–d). It should be emphasized that these enhancements are more prominent compared with the PA gel with relatively strong primary chain entanglements (*C*_M_ = 2.3 M) reported previously [45]. These significant improvements demonstrate the highly efficient synergistic effect of ionic and metal coordination bonds. The above result initially confirmed our proposed approach is not limited for a specific PA gel system.

**Figure 7:**
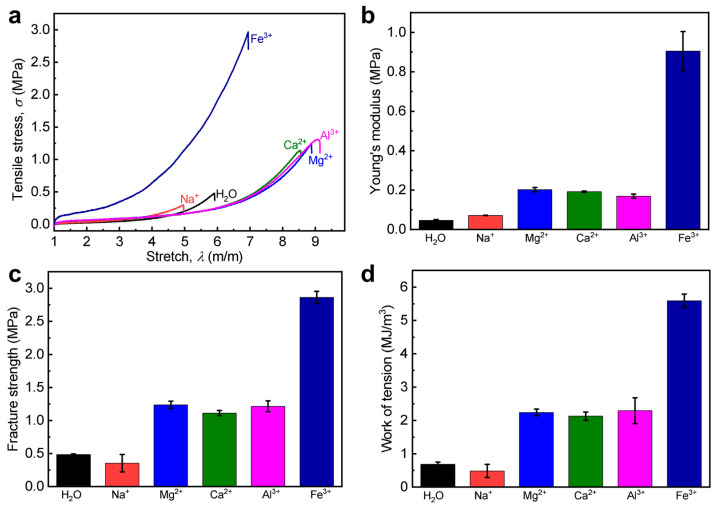
Tensile behavior of WEQ-PA-M hydrogels with different metal ions (M). Here the original PA gel is P(NaSS-*co*-DMAEA-Q) gel. M represents Fe^3+^, Al^3+^, Mg^2+^, Ca^2+^, and Na^+^ ions. (**a**) Tensile stress–stretch ratio curves. (**b**–**d**) Detailed Young’s modulus (**b**), tensile fracture strength (**c**), and work of tension (**d**). Values in (**b**–**d**) are expressed as mean value ± SD (*n* ≥ 3). *C*_M_ = 2.0 M, *C*_metal_ = 0.3 M.

To expand our understanding on the generality of the proposed approach, we further introduced different metal ions (i.e., Al^3+^, Mg^2+^, Ca^2+^, Na^+^) into the PA gel with a relatively low *C*_M_ (2.0 M) to prepare WEQ-PA-M gels, where M represents metal ions. The original PA gel is P(NaSS-*co*-DMAEA-Q) gel, and the concentration of the metal ion solution (*C*_metal_) for dialysis is 0.3 M. The tensile result demonstrates that the WEQ-PA-M gels with multivalent metal ions (i.e., Al^3+^, Mg^2+^, Ca^2+^) possess distinct mechanical improvements comparing with the neat gel, but the gel with monovalent ions (i.e., Na^+^) exhibits comparable mechanical performance due to the lack of coordination ability (Figure 7 and Table 3). The mechanical improvements of the gels with multivalent metal ions probably mainly result from the high synergy effectiveness of the formed ionic and metal coordination bonds, although the original gel possesses relatively weak primary chain entanglements at the as-prepared state. When further comparing the mechanical data among the WEQ-PA-M gels with different multivalent metal ions, we find that the mechanical enhancements of the gels with Fe^3+^ ions are the best, which should be mainly related to the binding parameters of the multivalent metal ions [45,59,60]. Thus, the result confirmed again that our proposed approach is relatively general for mechanically enhancing PA gels with relatively weak primary chain entanglements. All these data demonstrate that our proposed secondary equilibrium approach is extended successfully for the toughening of weak PA gels.

## 4. Conclusions

In summary, we have prepared strong and tough modified PA hydrogels with relatively weak primary chain entanglements via a secondary equilibrium approach. The chain entanglements could be controlled by the monomer concentration in the pre-gel solutions. According to the approach, an as-prepared PA gel was first dialyzed in a multivalent metal ion solution to reach a swelling equilibrium (Step-I) and then soaked in deionized water to dialyze out excess free ions to achieve a new equilibrium (Step-II), resulting in the modified PA gel. Through this approach, it was proved that the PA gel with relatively weak primary chain entanglements could be toughened effectively by the synergy of the formed ionic and metal coordination bonds. The effects of chain entanglements and metal ion concentration on the mechanical properties of the gels were systematically studied by varying *C*_M_ (1.5–2.5 M) (in the pre-gel solutions) and $C_{{\mathrm{FeCl}}_{3}}$ (0–2.0 M) (in Step-I). The result demonstrated that the PA gels with different *C*_M_ could be mechanically enhanced effectively, and the enhancements were maximized at *C*_M_ = 2.0 M. In addition, different $C_{{\mathrm{FeCl}}_{3}}$ also resulted in different mechanical enhancements, and the synergy between ionic and metal coordination bonds is the most effective at $C_{{\mathrm{FeCl}}_{3}}$ = 0.3 M. The modified PA gel could achieve the optimal mechanical performance at *C*_M_ = 2.0 M and $C_{{\mathrm{FeCl}}_{3}}$ = 0.3 M: its elastic modulus, tensile fracture strength, and work of tension were improved by 1800%, 600%, and 820%, respectively, comparing to the original PA gel. By selecting different PA gel system and diverse multivalent metal ions (i.e., Al^3+^, Mg^2+^, Ca^2+^), our approach was proved to be relatively general. Further studies (e.g., biocompatibility, physiological stability, and cell cytotoxicity) are needed in future work to explore the potential applications of the gels. This study well extends our proposed secondary equilibrium approach for the toughening of weak PA gels with relatively weak chain entanglements.

## Figures and Tables

**Table 1:** Summary of tensile properties of WEQ-PA-Fe gels. Here the original PA gel is P(NaSS-*co*-DMAEA-Q) gel.

$\mathrm{Sample}\;\mathrm{Code}\;(C_{M}-C_{{\mathrm{FeCl}}_{3}}$) ^(a)^	*E* (MPa)	*ε*_b_ (m m^−1^)	*σ*_b_ (MPa)	*W*_b_ (MJ m^−3^)	*Q*_v_ (m^3^ m^−3^)
WEQ-PA-1.5-Fe-0.3	0.17 ± 0.01	8.0 ± 0.1	1.4 ± 0.1	3.6 ± 0.3	0.52 ± 0.01
WEQ-PA-1.7-Fe-0.3	0.48 ± 0.01	6.7 ± 0.1	2.2 ± 0.2	4.7 ± 0.6	0.62 ± 0.02
WEQ-PA-2.0-Fe-0.3	0.90 ± 0.09	6.1 ± 0.1	2.9 ± 0.1	5.6 ± 0.2	0.57 ± 0.01
WEQ-PA-2.3-Fe-0.3	0.23 ± 0.01	6.0 ± 0.1	2.3 ± 0.2	4.3 ± 0.6	0.70 ± 0.01
WEQ-PA-2.5-Fe-0.3	0.26 ± 0.01	6.0 ± 0.1	1.7 ± 0.1	3.2 ± 0.4	0.70 ± 0.01
WEQ-PA-2.0	0.05 ± 0.01	5.1 ± 0.1	0.48 ± 0.01	0.68 ± 0.06	0.94 ± 0.06
WEQ-PA-2.0-Fe-0.01	0.53 ± 0.04	6.0 ± 0.1	2.3 ± 0.1	4.1 ± 0.1	0.59 ± 0.01
WEQ-PA-2.0-Fe-0.02	0.52 ± 0.03	5.9 ± 0.1	2.4 ± 0.1	4.0 ± 0.2	0.61 ± 0.01
WEQ-PA-2.0-Fe-0.05	0.59 ± 0.01	6.0 ± 0.1	2.4 ± 0.3	4.6 ± 0.7	0.58 ± 0.01
WEQ-PA-2.0-Fe-0.1	0.80 ± 0.05	6.1 ± 0.1	2.7 ± 0.1	5.2 ± 0.3	0.58 ± 0.01
WEQ-PA-2.0-Fe-0.5	0.72 ± 0.05	6.0 ± 0.1	2.5 ± 0.1	4.9 ± 0.6	0.60 ± 0.02
WEQ-PA-2.0-Fe-0.7	0.58 ± 0.01	6.0 ± 0.1	2.2 ± 0.1	4.5 ± 0.1	0.60 ± 0.01
WEQ-PA-2.0-Fe-1.0	0.51 ± 0.03	6.1 ± 0.1	2.2 ± 0.2	4.3 ± 0.4	0.61 ± 0.02
WEQ-PA-2.0-Fe-1.5	0.52 ± 0.03	6.0 ± 0.1	2.2 ± 0.1	4.4 ± 0.2	0.61 ± 0.02
WEQ-PA-2.0-Fe-2.0	0.52 ± 0.02	6.2 ± 0.1	2.4 ± 0.1	4.9 ± 0.4	0.60 ± 0.03

^(a)^ *C*_M_ in the code of WEQ-PA-*C*_M_-Fe-$C_{{\mathrm{FeCl}}_{3}}$ represents the monomer concentration in pre-gel solutions.

**Table 2:** Summary of tensile properties of WEQ-PA-Fe hydrogels. Here the original PA gel is P(NaSS-*co*-MPTC) gel.

$\mathrm{Sample}\;\mathrm{Code}\;(C_{M}-C_{{\mathrm{FeCl}}_{3}}$) ^(a)^	*E* (MPa)	*ε*_b_ (m m^−1^)	*σ*_b_ (MPa)	*W*_b_ (MJ m^−3^)	*Q*_v_ (m^3^ m^−3^)
WEQ-PA-1.5	0.02 ± 0.01	0.82 ± 0.02	0.01 ± 0.01	0.007 ± 0.001	4.8 ± 0.2
WEQ-PA-1.5-Fe-0.01	7.0 ± 0.3	2.7 ± 0.1	1.3 ± 0.1	2.5 ± 0.1	0.68 ± 0.01
WEQ-PA-1.5-Fe-0.02	6.6 ± 0.2	2.7 ± 0.1	1.4 ± 0.1	2.5 ± 0.2	0.67 ± 0.01
WEQ-PA-1.5-Fe-0.05	6.5 ± 0.4	3.1 ± 0.1	1.5 ± 0.1	2.9 ± 0.1	0.66 ± 0.01
WEQ-PA-1.5-Fe-0.1	6.3 ± 0.2	2.8 ± 0.1	1.2 ± 0.1	2.4 ± 0.1	0.67 ± 0.01
WEQ-PA-1.5-Fe-0.3	2.6 ± 0.1	2.6 ± 0.1	0.55 ± 0.04	0.87 ± 0.13	1.1 ± 0.1
WEQ-PA-1.5-Fe-0.5	1.6 ± 0.2	2.6 ± 0.1	0.42 ± 0.02	0.68 ± 0.09	1.3 ± 0.1
WEQ-PA-1.5-Fe-0.7	1.1 ± 0.2	2.6 ± 0.1	0.34 ± 0.01	0.55 ± 0.05	1.4 ± 0.1
WEQ-PA-1.5-Fe-1.0	1.0 ± 0.2	2.0 ± 0.1	0.24 ± 0.01	0.30 ± 0.01	1.6 ± 0.1
WEQ-PA-1.5-Fe-1.5	1.0 ± 0.1	2.0 ± 0.1	0.26 ± 0.01	0.34 ± 0.03	1.5 ± 0.1

^(a)^ *C*_M_ in the code of WEQ-PA-*C*_M_-Fe-$C_{{\mathrm{FeCl}}_{3}}$ represents the monomer concentration in pre-gel solutions.

**Table 3:** Summary of tensile properties of WEQ-PA-M hydrogels. Here the original PA gel is P(NaSS-*co*-DMAEA-Q) gel.

$\mathrm{Sample}\;\mathrm{Code}\;(C_{M}-C_{\mathrm{metal}}$) ^(a)^	*E* (MPa)	*ε*_b_ (m m^−1^)	*σ*_b_ (MPa)	*W*_b_ (MJ m^−3^)	*Q*_v_ (m^3^ m^−3^)
WEQ-PA-2.0	0.05 ± 0.01	5.1 ± 0.1	0.48 ± 0.01	0.68 ± 0.06	0.94 ± 0.06
WEQ-PA-2.0-Fe-0.3	0.91 ± 0.09	6.1 ± 0.1	2.9 ± 0.1	5.6 ± 0.2	0.57 ± 0.01
WEQ-PA-2.0-Al-0.3	0.17 ± 0.01	7.9 ± 0.2	1.2 ± 0.1	2.3 ± 0.4	0.69 ± 0.01
WEQ-PA-2.0-Mg-0.3	0.20 ± 0.01	7.7 ± 0.2	1.2 ± 0.1	2.2 ± 0.1	0.74 ± 0.01
WEQ-PA-2.0-Ca-0.3	0.19 ± 0.01	7.5 ± 0.2	1.1 ± 0.1	2.1 ± 0.1	0.77 ± 0.02
WEQ-PA-2.0-Na-0.3	0.071 ± 0.002	4.0 ± 0.1	0.35 ± 0.13	0.48 ± 0.02	0.96 ± 0.03

^(a)^ *C*_M_ in the code of WEQ-PA-*C*_M_-M-$C_{\mathrm{metal}}$ represents the monomer concentration in pre-gel solutions.

## Data Availability

Not applicable.

## Supplementary Materials

The following supporting information can be downloaded at: https://www.mdpi.com/article/10.3390/polym15122644/s1, Figure S1: Possible dynamic bonds formed in the WEQ-PA-Fe hydrogel networks; Figure S2: Hydrogel sample geometry and method for tensile tests; Figure S3: Water content (*ω*_w_) of ASP-PA gels with different *C*_M_ (1.5–2.5 M); Figure S4: FTIR spectra of dried WEQ-PA and WEQ-PA-Fe hydrogels; Figure S5: Comparison of tensile properties of WEQ-PNA gel, WEQ-PNA-Fe gel, WEQ-PA gel, and WEQ-PA-Fe gel; Figure S6: FTIR spectra of dried WEQ-PA-Fe hydrogels with different initial monomer concentration, *C*_M_.; Figure S7: FTIR spectra of dried WEQ-PA-Fe hydrogels with different $C_{{\mathrm{FeCl}}_{3}}$; Table S1: Atomic ratio of Fe:S in WEQ-*n*-PA-Fe gels on the basis of the XRF result; Table S2: Summary of tensile properties of WEQ-PNA gel, WEQ-PNA-Fe gel, WEQ-PA gel, and WEQ-PA-Fe gel shown in Figure S5; Table S3: Summary of tensile properties of neat WEQ-PA gels with different *C*_M_ (1.5–2.5 M). References [52,61] are cited in the supplementary materials.

## References

[1] Zhang Y.S., Khademhosseini A. (2017). Advances in Engineering Hydrogels. Science.

[2] Huang Y., Zeng M., Ren J., Wang J., Fan L., Xu Q. (2012). Preparation and Swelling Properties of Graphene Oxide/Poly(Acrylic Acid-Co-Acrylamide) Super-Absorbent Hydrogel Nanocomposites. Colloids Surf. A Physicochem. Eng. Asp..

[3] Wei H., Lei M., Zhang P., Leng J., Zheng Z., Yu Y. (2021). Orthogonal Photochemistry-Assisted Printing of 3D Tough and Stretchable Conductive Hydrogels. Nat. Commun..

[4] Deng J., Wu H., Xie W., Jia H., Xia Z., Wang H. (2021). Metal Cation-Responsive and Excitation-Dependent Nontraditional Multicolor Fluorescent Hydrogels for Multidimensional Information Encryption. ACS Appl. Mater. Interfaces.

[5] Wang C., Chen X., Wang L., Makihata M., Liu H.-C., Zhou T., Zhao X. (2022). Bioadhesive Ultrasound for Long-Term Continuous Imaging of Diverse Organs. Science.

[6] Ma Z., Bourquard C., Gao Q., Jiang S., De Iure-Grimmel T., Huo R., Li X., He Z., Yang Z., Yang G. (2022). Controlled Tough Bioadhesion Mediated by Ultrasound. Science.

[7] Na H., Kang Y.-W., Park C.S., Jung S., Kim H.-Y., Sun J.-Y. (2022). Hydrogel-Based Strong and Fast Actuators by Electroosmotic Turgor Pressure. Science.

[8] Li C.Y., Zheng S.Y., Hao X.P., Hong W., Zheng Q., Wu Z.L. (2022). Spontaneous and Rapid Electro-Actuated Snapping of Constrained Polyelectrolyte Hydrogels. Sci. Adv..

[9] Sun Y., Le X., Zhou S., Chen T. (2022). Recent Progress in Smart Polymeric Gel-Based Information Storage for Anti-Counterfeiting. Adv. Mater..

[10] Liang X., Chen G., Lin S., Zhang J., Wang L., Zhang P., Lan Y., Liu J. (2022). Bioinspired 2D Isotropically Fatigue-Resistant Hydrogels. Adv. Mater..

[11] Jiang Y., Zhang X., Zhang W., Wang M., Yan L., Wang K., Han L., Lu X. (2022). Infant Skin Friendly Adhesive Hydrogel Patch Activated at Body Temperature for Bioelectronics Securing and Diabetic Wound Healing. ACS Nano.

[12] Bian X., Cui C., Qi Y., Sun Y., Zhang Z., Liu W. (2022). Amino Acid Surfactant-Induced Superfast Gelation of Silk Fibroin for Treating Noncompressible Hemorrhage. Adv. Funct. Mater..

[13] Huang Y., Mu L., Zhao X., Han Y., Guo B. (2022). Bacterial Growth-Induced Tobramycin Smart Release Self-Healing Hydrogel for Pseudomonas Aeruginosa -Infected Burn Wound Healing. ACS Nano.

[14] Li Y., Liu L., Xu H., Cheng Z., Yan J., Xie X.-M. (2022). Biomimetic Gradient Hydrogel Actuators with Ultrafast Thermo-Responsiveness and High Strength. ACS Appl. Mater. Interfaces.

[15] Chen G., Wang K., Yang J., Huang J., Chen Z., Zheng J., Wang J., Yang H., Li S., Miao Y. (2023). Printable Thermochromic Hydrogel-Based Smart Window for All-Weather Building Temperature Regulation in Diverse Climates. Adv. Mater..

[16] Zhong R., Talebian S., Mendes B.B., Wallace G., Langer R., Conde J., Shi J. (2023). Hydrogels for RNA Delivery. Nat. Mater..

[17] Zhang Y., Xu Z., Yuan Y., Liu C., Zhang M., Zhang L., Wan P. (2023). Flexible Antiswelling Photothermal-Therapy MXene Hydrogel-Based Epidermal Sensor for Intelligent Human–Machine Interfacing. Adv. Funct. Mater..

[18] He H., Li H., Pu A., Li W., Ban K., Xu L. (2023). Hybrid Assembly of Polymeric Nanofiber Network for Robust and Electronically Conductive Hydrogels. Nat. Commun..

[19] Xiong X., Chen Y., Wang Z., Liu H., Le M., Lin C., Wu G., Wang L., Shi X., Jia Y.-G. (2023). Polymerizable Rotaxane Hydrogels for Three-Dimensional Printing Fabrication of Wearable Sensors. Nat. Commun..

[20] Peng L., Hou L., Wu P. (2023). Synergetic Lithium and Hydrogen Bonds Endow Liquid-Free Photonic Ionic Elastomer with Mechanical Robustness and Electrical/Optical Dual-Output. Adv. Mater..

[21] Long S., Huang J., Xiong J., Liu C., Chen F., Shen J., Huang Y., Li X. (2023). Designing Multistimuli-Responsive Anisotropic Bilayer Hydrogel Actuators by Integrating LCST Phase Transition and Photochromic Isomerization. Polymers.

[22] Gong J.P. (2010). Why Are Double Network Hydrogels so Tough?. Soft Matter.

[23] Gong J.P., Katsuyama Y., Kurokawa T., Osada Y. (2003). Double-Network Hydrogels with Extremely High Mechanical Strength. Adv. Mater..

[24] Sakai T., Matsunaga T., Yamamoto Y., Ito C., Yoshida R., Suzuki S., Sasaki N., Shibayama M., Chung U. (2008). Il Design and Fabrication of a High-Strength Hydrogel with Ideally Homogeneous Network Structure from Tetrahedron-like Macromonomers. Macromolecules.

[25] Liu C., Morimoto N., Jiang L., Kawahara S., Noritomi T., Yokoyama H., Mayumi K., Ito K. (2021). Tough Hydrogels with Rapid Self-Reinforcement. Science.

[26] Matsuda T., Kawakami R., Namba R., Nakajima T., Gong J.P. (2019). Mechanoresponsive Self-Growing Hydrogels Inspired by Muscle Training. Science.

[27] Huang Y., Xiao L., Zhou J., Li X., Liu J., Zeng M. (2020). Mechanical Enhancement of Graphene Oxide-Filled Chitosan-Based Composite Hydrogels by Multiple Mechanisms. J. Mater. Sci..

[28] Yan Y., Xiao L., Teng Q., Jiang Y., Deng Q., Li X., Huang Y. (2022). Strong, Tough, and Adhesive Polyampholyte/Natural Fiber Composite Hydrogels. Polymers.

[29] Zhang M., Yang Y., Li M., Shang Q., Xie R., Yu J., Shen K., Zhang Y., Cheng Y. (2023). Toughening Double Network Hydrogels by Polyelectrolytes. Adv. Mater..

[30] Huang Y., King D.R., Sun T.L., Nonoyama T., Kurokawa T., Nakajima T., Gong J.P. (2017). Energy-Dissipative Matrices Enable Synergistic Toughening in Fiber Reinforced Soft Composites. Adv. Funct. Mater..

[31] Shi Y., Wu B., Sun S., Wu P. (2023). Aqueous Spinning of Robust, Self-Healable, and Crack-Resistant Hydrogel Microfibers Enabled by Hydrogen Bond Nanoconfinement. Nat. Commun..

[32] Hou L.X., Ju H., Hao X.P., Zhang H., Zhang L., He Z., Wang J., Zheng Q., Wu Z.L. (2023). Intrinsic Anti-Freezing and Unique Phosphorescence of Glassy Hydrogels with Ultrahigh Stiffness and Toughness at Low Temperatures. Adv. Mater..

[33] Xu L., Qiao Y., Qiu D. (2023). Coordinatively Stiffen and Toughen Hydrogels with Adaptable Crystal-Domain Cross-Linking. Adv. Mater..

[34] Ji D., Park J.M., Oh M.S., Nguyen T.L., Shin H., Kim J.S., Kim D., Park H.S., Kim J. (2022). Superstrong, Superstiff, and Conductive Alginate Hydrogels. Nat. Commun..

[35] Lian W.Z., Fan Z.W., Cui K., Yin P., Yang J., Jiang H., Tang L., Sun T. (2021). Tough Hydrogels with Dynamic H-Bonds: Structural Heterogeneities and Mechanical Performances. Macromolecules.

[36] Cao J., Li J., Chen Y., Zhang L., Zhou J. (2018). Dual Physical Crosslinking Strategy to Construct Moldable Hydrogels with Ultrahigh Strength and Toughness. Adv. Funct. Mater..

[37] Hua M., Wu S., Ma Y., Zhao Y., Chen Z., Frenkel I., Strzalka J., Zhou H., Zhu X., He X. (2021). Strong Tough Hydrogels via the Synergy of Freeze-Casting and Salting Out. Nature.

[38] Du C., Zhang X.N., Sun T.L., Du M., Zheng Q., Wu Z.L. (2021). Hydrogen-Bond Association-Mediated Dynamics and Viscoelastic Properties of Tough Supramolecular Hydrogels. Macromolecules.

[39] Cui W., Zheng Y., Zhu R., Mu Q., Wang X., Wang Z., Liu S., Li M., Ran R. (2022). Strong Tough Conductive Hydrogels via the Synergy of Ion-Induced Cross-Linking and Salting-Out. Adv. Funct. Mater..

[40] Wu J., Pan Z., Zhao Z., Wang M., Dong L., Gao H., Liu C., Zhou P., Chen L., Shi C. (2022). Anti-Swelling, Robust, and Adhesive Extracellular Matrix-Mimicking Hydrogel Used as Intraoral Dressing. Adv. Mater..

[41] Xu J., Jin R., Ren X., Gao G. (2019). Cartilage-Inspired Hydrogel Strain Sensors with Ultrahigh Toughness, Good Self-Recovery and Stable Anti-Swelling Properties. J. Mater. Chem. A.

[42] Zhang X.N., Du C., Wang Y.J., Hou L.X., Du M., Zheng Q., Wu Z.L. (2022). Influence of the α-Methyl Group on Elastic-To-Glassy Transition of Supramolecular Hydrogels with Hydrogen-Bond Associations. Macromolecules.

[43] Huang G., Tang Z., Peng S., Zhang P., Sun T., Wei W., Zeng L., Guo H., Guo H., Meng G. (2022). Modification of Hydrophobic Hydrogels into a Strongly Adhesive and Tough Hydrogel by Electrostatic Interaction. Macromolecules.

[44] Zhou J., Huang Y., Qian S., Zeng P., Long S., Li X. (2022). Strengthening and Stiffening in Swollen Polyampholyte Hydrogels. Mater. Lett..

[45] Huang Y., Xiao L., Zhou J., Liu T., Yan Y., Long S., Li X. (2021). Strong Tough Polyampholyte Hydrogels via the Synergistic Effect of Ionic and Metal–Ligand Bonds. Adv. Funct. Mater..

[46] Sun J.-Y., Zhao X., Illeperuma W.R.K., Chaudhuri O., Oh K.H., Mooney D.J., Vlassak J.J., Suo Z. (2012). Highly Stretchable and Tough Hydrogels. Nature.

[47] Sun T.L., Kurokawa T., Kuroda S., Ihsan A.B., Akasaki T., Sato K., Haque M.A., Nakajima T., Gong J.P. (2013). Physical Hydrogels Composed of Polyampholytes Demonstrate High Toughness and Viscoelasticity. Nat. Mater..

[48] Ihsan A.B., Sun T.L., Kuroda S., Haque M.A., Kurokawa T., Nakajima T., Gong J.P. (2013). A Phase Diagram of Neutral Polyampholyte—From Solution to Tough Hydrogel. J. Mater. Chem. B.

[49] Cui K., Sun T.L., Liang X., Nakajima K., Ye Y.N., Chen L., Kurokawa T., Gong J.P. (2018). Multiscale Energy Dissipation Mechanism in Tough and Self-Healing Hydrogels. Phys. Rev. Lett..

[50] Li X., Cui K., Kurokawa T., Ye Y.N., Sun T.L., Yu C., Creton C., Gong J.P. (2021). Effect of Mesoscale Phase Contrast on Fatigue-Delaying Behavior of Self-Healing Hydrogels. Sci. Adv..

[51] Huang Y., King D.R., Cui W., Sun T.L., Guo H., Kurokawa T., Brown H.R., Hui C.-Y., Gong J.P. (2019). Superior Fracture Resistance of Fiber Reinforced Polyampholyte Hydrogels Achieved by Extraordinarily Large Energy-Dissipative Process Zones. J. Mater. Chem. A.

[52] Huang Y., Qian S., Zhou J., Chen W., Liu T., Yang S., Long S., Li X. (2023). Achieving Swollen yet Strengthened Hydrogels by Reorganizing Multiphase Network Structure. Adv. Funct. Mater..

[53] Obukhov S.P., Rubinstein M., Colby R.H. (1994). Network Modulus and Superelasticity. Macromolecules.

[54] Sun T.L., Luo F., Kurokawa T., Karobi S.N., Nakajima T., Gong J.P. (2015). Molecular Structure of Self-Healing Polyampholyte Hydrogels Analyzed from Tensile Behaviors. Soft Matter.

[55] Xiao S., Zhang Y., Shen M., Chen F., Fan P., Zhong M., Ren B., Yang J., Zheng J. (2018). Structural Dependence of Salt-Responsive Polyzwitterionic Brushes with an Anti-Polyelectrolyte Effect. Langmuir.

[56] Lin W.C., Fan W., Marcellan A., Hourdet D., Creton C. (2010). Large Strain and Fracture Properties of Poly(Dimethylacrylamide)/Silica Hybrid Hydrogels. Macromolecules.

[57] Wang T., Liu D., Lian C., Zheng S., Liu X., Tong Z. (2012). Large Deformation Behavior and Effective Network Chain Density of Swollen Poly(N-Isopropylacrylamide)–Laponite Nanocomposite Hydrogels. Soft Matter.

[58] Nian G., Kim J., Bao X., Suo Z. (2022). Making Highly Elastic and Tough Hydrogels from Doughs. Adv. Mater..

[59] Yu H.C., Li C.Y., Du M., Song Y., Wu Z.L., Zheng Q. (2019). Improved Toughness and Stability of *κ*-Carrageenan/Polyacrylamide Double-Network Hydrogels by Dual Cross-Linking of the First Network. Macromolecules.

[60] Shannon R.D. (1976). Revised Effective Ionic Radii and Systematic Studies of Interatomic Distances in Halides and Chalcogenides. Acta Crystallogr. Sect. A Found. Crystallogr..

[61] Li Y., Wen J., Qin M., Cao Y., Ma H., Wang W. (2017). Single-Molecule Mechanics of Catechol-Iron Coordination Bonds. ACS Biomater. Sci. Eng..

