# Supplementary material for: Toughening Weak Polyampholyte Hydrogels with Weak Chain Entanglements via a Secondary Equilibrium Approach
Source: Polymers (Basel). 2023 Jun 10;15(12):2644. doi: 10.3390/polym15122644 (PMC10301078; doi:10.3390/polym15122644)
Supplement: Supplementary file 1 [file polymers-15-02644-s001.zip › polymers-2437880-supplementary.pdf]

Supplementary Materials

Toughening Weak Polyampholyte Hydrogels with Weak Chain Entanglements via a Secondary Equilibrium Approach

Tao Liu [1,†], Wenjun Chen [1,†], Kai Li [1], Shijun Long [1,2], Xuefeng Li [1,2] and Yiwan Huang [1,2,3,4,*]

[1] Hubei Provincial Key Laboratory of Green Materials for Light Industry, Hubei University of Technology, Wuhan 430068, China
[2] New Materials and Green Manufacturing Talent Introduction and Innovation Demonstration Base, Hubei University of Technology, Wuhan 430068, China
[3] Non-Power Nuclear Technology Collaborative Innovation Center, Hubei University of Science and Technology, Xianning 437100, China
[4] Hubei Longzhong Laboratory, Xiangyang 441000, China
* Correspondence: yiwanhuang@hbut.edu.cn
† The authors contributed equally to this work.

S1. Supplementary Methods

S1.1. Fourier Transform Infrared Spectroscopy (FTIR)

FTIR spectra of the hydrogel samples were recorded by using an FTIR spectrometer (Bruker, Tensor II). Before measurements, the samples were vacuum-dried for 48 h. The specimens were prepared by the KBr-disk method, and the spectra in the wavenumber range of 4000–400 cm^{-1} were collected with a resolution of 4 cm^{-1}. All the experiments were carried out at ambient temperature.

S1.2. Water Content of Hydrogels

The water content of (ω_w) hydrogel samples was measured by electronic moisture meter (MOC-120H, Shimadzu). Before measurement, cut more than 0.5 g of hydrogel samples and gently wipe the balanced wet samples with filter paper to remove water on the surface; The temperature of the electronic moisture meter is set to 120 ºC until the moisture change of the electronic moisture meter is less than 0.01% within a period of time.

S1.3. X-ray Fluorescence Spectrometer (XRF)

The element content inside the hydrogel samples was evaluated by energy dispersive X-ray fluorescence spectrometer (XRF, EDX-8100, Shimadzu). Before measurements, hydrogel samples with appropriate size were wiped out to remove the water on the surface. During the measurements, the samples were excited by the incident X-ray generated through the X-ray tube. Each element in a stimulated sample would emit secondary X-rays, and the secondary X-rays emitted by different elements have specific energy characteristics or wavelength characteristics. The detection system could detect the information collected by these systems and convert it into the contents of various elements in the sample.

S2. Supplementary Figures

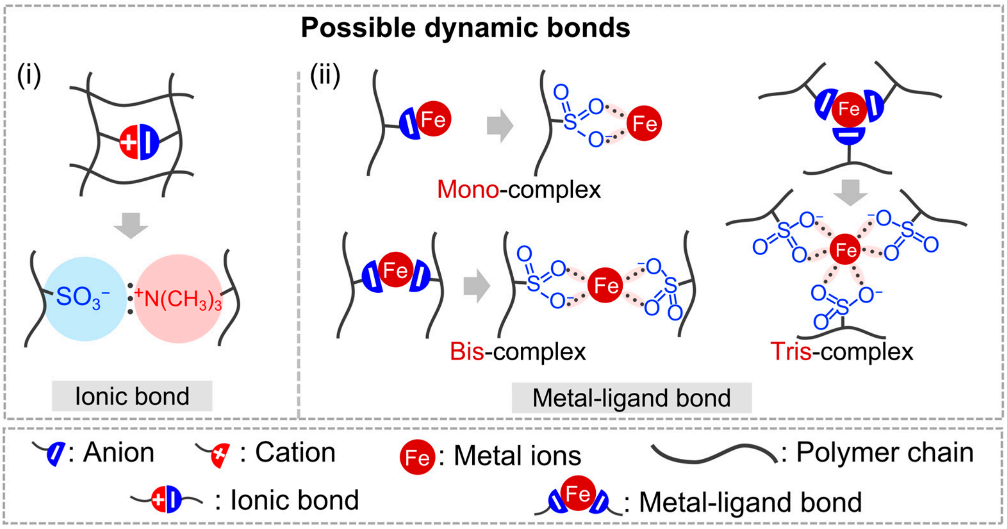

Figure **S1.** Possible dynamic bonds formed in the WEQ-PA-Fe hydrogel networks.

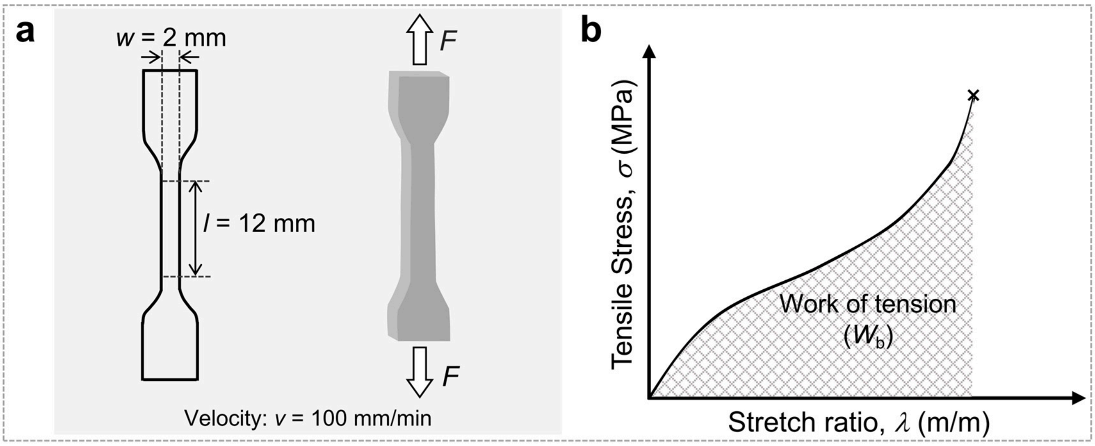

Figure S2. Hydrogel sample geometry and method for tensile tests. (**a**) Geometry of tensile tests. The samples were cut into a dumbbell shape (standard: JIS-K6251-7) (thickness: t = 1–2 mm) prior to the tests. (**b**) Calculation of work of tension (W_b) by integrating the area under the stress-stretch curve.

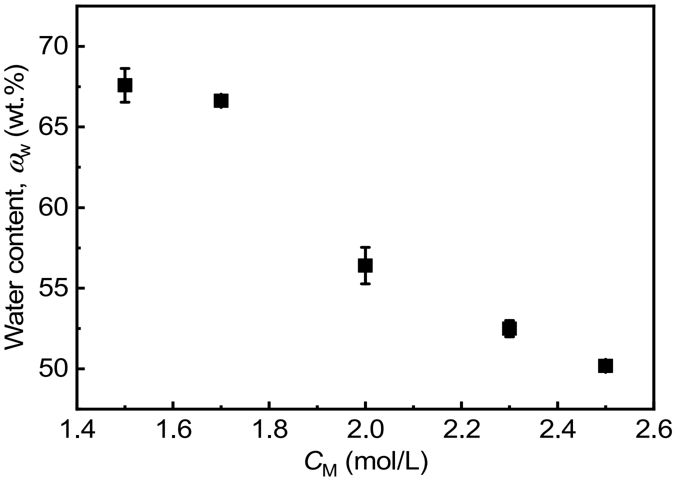

Figure S3. Water content (ω_w) of ASP-PA gels with different C_M (1.5–2.5 M).

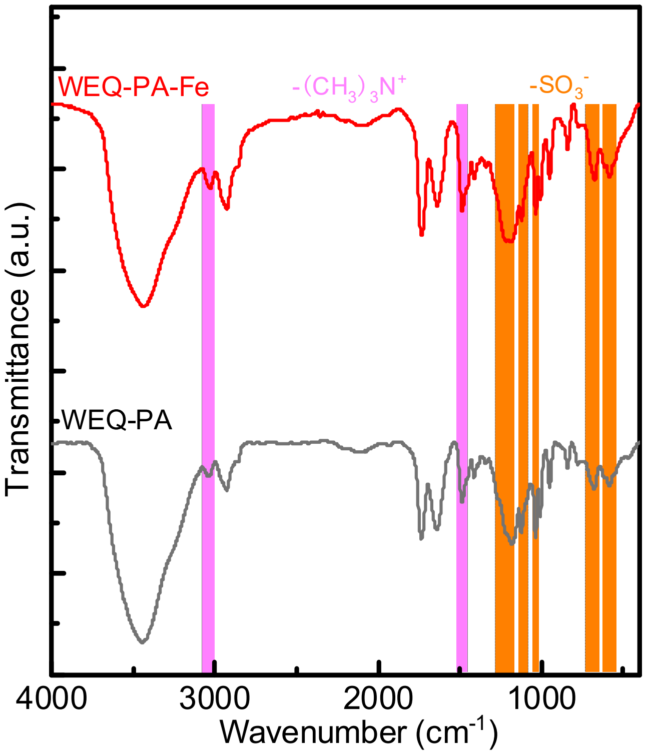

Figure S4. FTIR spectra of dried WEQ-PA and WEQ-PA-Fe hydrogels. Here the original PA gel is P(NaSS-*co*-DMAEA-Q) gel. C_M = 0.3 M. C_{FeCl_3} = 0.3 M. In the spectrum of WEQ-PA gel, the vibration peaks at 1186, 1122, 1036, 677, and 585 cm^{-1} are correlated to $-SO_3^-$ groups, and the peaks at 3039 and 1487 cm^{-1} are correlated to $-(CH_3)_3N^+$ (i.e., −C−N) groups on the polymer chains. After the introduction of Fe^{3+} ions, almost all characteristic peaks of $-SO_3^-$ groups redshift and become stronger and broader in WEQ-PA-Fe gel, suggesting the formation of metal-coordination ($Fe^{3+}\cdots{}^-SO_3-$) bonds. Additionally, the characteristic peaks of $-(CH_3)_3N^+$ groups also become stronger and broader in WEQ-PA-Fe gel, indicating the strengthening of the ionic bonds between the oppositely charged groups.

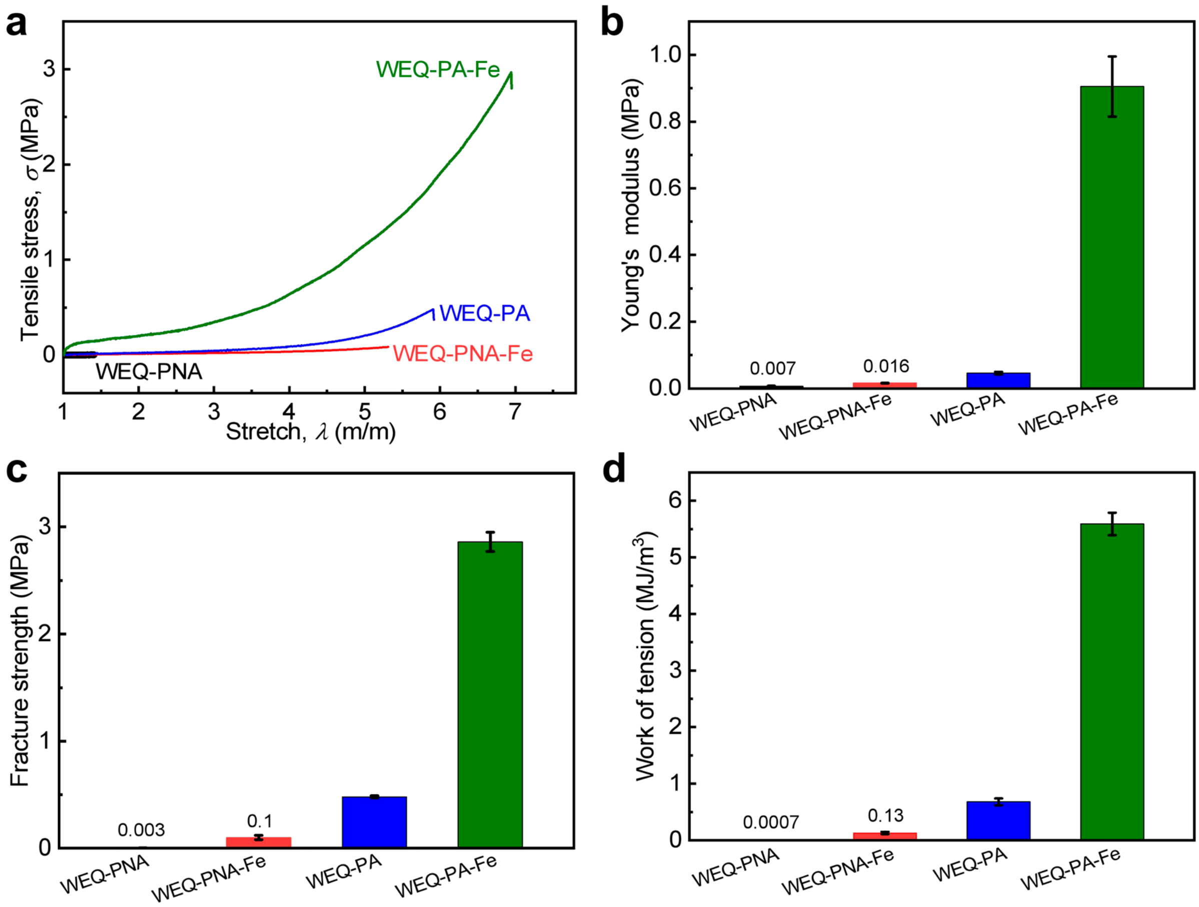

Figure S5. Comparison of tensile properties of WEQ-PNA gel, WEQ-PNA-Fe gel, WEQ-PA gel, and WEQ-PA-Fe gel. (**a**) Tensile stress-stretch curves. (**b**) Young's modulus. (**c**) Tensile fracture strength. (**d**) Work of tension. The original PA gel is non-neutral P(NaSS-*co*-DMAEA-Q) gel, and the original PNA gel is P(NaSS-*co*-AAm) gel. C_M= 2.0 M, C_{FeCl_3}= 0.3 M.

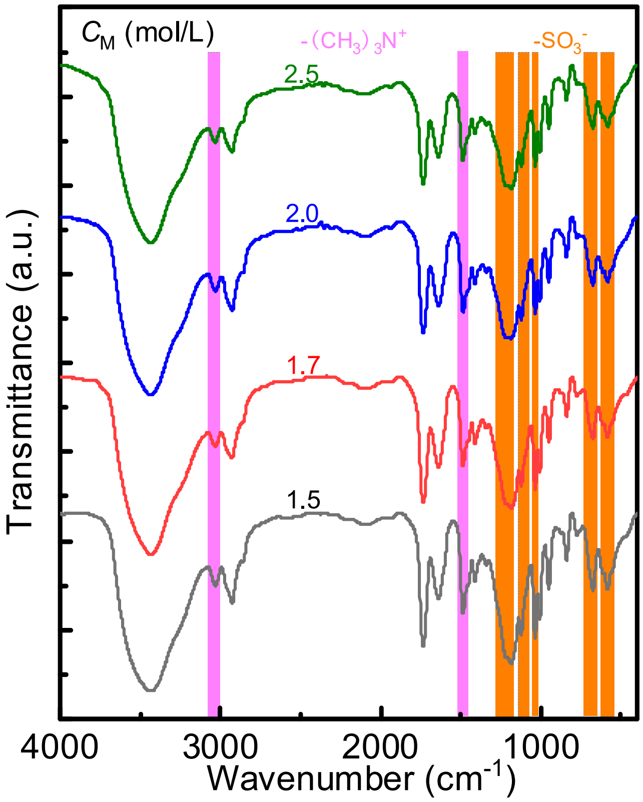

Figure S6. FTIR spectra of dried WEQ-PA-Fe hydrogels with different initial monomer concentration, C_M. Here the original PA gel is P(NaSS-*co*-DMAEA-Q) gel. C_{FeCl_3}= 0.3 M. The relatively strong characteristic peaks of both the $-SO_3^-$ and $-(CH_3)_3N^+$ (i.e., −C−N) groups indicate the effective formation of both metal-coordination (Fe^{3+}•••$^-SO_3$–) bonds and ionic bonds, synergistically enabling the mechanical enhancements of WEQ-PA-Fe hydrogels [52].

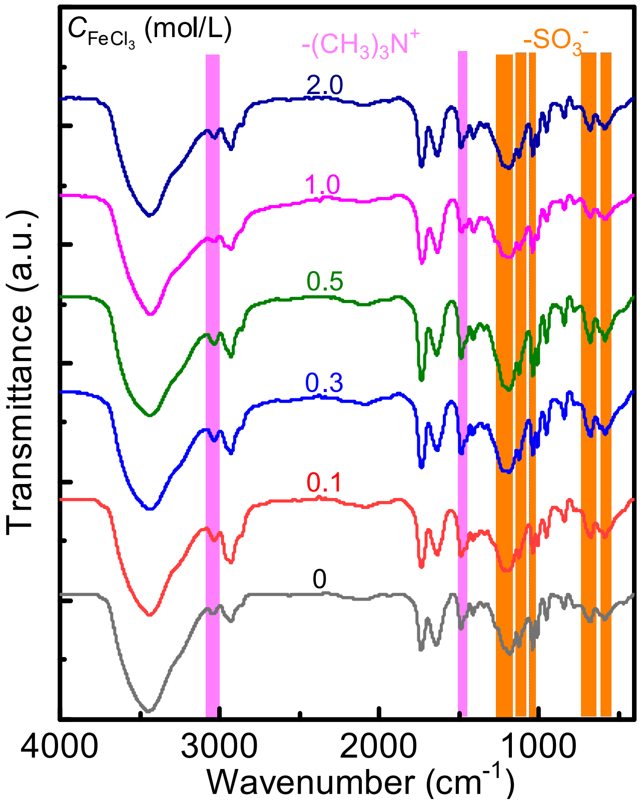

Figure S7. FTIR spectra of dried WEQ-PA-Fe hydrogels with different C_{FeCl_3}. Here the original PA gel is P(NaSS-*co*-DMAEA-Q) gel. $C_M = 2.0$ M. The characteristic peaks of both the $-SO_3^-$ and $-(CH_3)_3N^+$ (i.e., −C−N) groups become relatively stronger at $0 < C_{FeCl_3} \leq 0.1$ M, then become comparable at $0.1 < C_{FeCl_3} \leq 0.5$ M, and become relatively weaker after $C_{FeCl_3} \geq 0.5$ M. The result indicates that the chemical structures of the gels were influenced by C_{FeCl_3} by affecting the quantity and quality of the formed ionic and metal-coordination bonds.

S3. Supplementary Tables

Table S1. Atomic ratio of Fe:S in WEQ-*n*-PA-Fe gels on the basis of the XRF result. Here the original PA gel is P(NaSS-*co*-DMAEA-Q) gel. C_M = 2.0 M.

Sample code (C_{FeCl_3})	Mass percentage (wt.%)		Atomic ratio	n(metal-coordination): n(ionic)[a]		
	Fe	S	Fe:S	Mono-complex[b]	Bis-complex[b]	Tris-complex[b]
WEQ-PA-Fe-0.01	0.44	15.08	0.017	0.017	0.034	0.053
WEQ-PA-Fe-0.02	0.68	16.44	0.024	0.024	0.050	0.076
WEQ-PA-Fe-0.05	1.46	17.81	0.047	0.049	0.103	0.164
WEQ-PA-Fe-0.1	1.89	17.52	0.062	0.066	0.141	0.227
WEQ-PA-Fe-0.3	4.61	16.87	0.156	0.185	0.454	0.881
WEQ-PA-Fe-0.5	5.11	16.35	0.179	0.217	0.556	1.154
WEQ-PA-Fe-0.7	5.64	16.12	0.200	0.250	0.666	1.499
WEQ-PA-Fe-1.0	5.66	15.83	0.204	0.257	0.691	1.584
WEQ-PA-Fe-1.5	5.31	15.56	0.195	0.242	0.639	1.410
WEQ-PA-Fe-2.0	5.72	15.21	0.215	0.274	0.754	1.814

[a] n(metal-coordination): n(ionic) is the hypothesized molar ratio of metal-coordination and ionic bonds in the hydrogels based on different coordination patterns. [b] Mono-, bis-, and tris-complexes mean three kinds of metal-coordination patterns between Fe^{3+} ions and $-SO_3^-$ groups.

The content of Fe element (ω_{Fe}) and atomic ratio of Fe:S ($r_{Fe/S}$) in WEQ-PA-Fe gels were measured by XRF and shown in **Table S1**. The result shows that ω_{Fe} and $r_{Fe/S}$ vary at 0.44–5.72 wt.% and 0.017–0.215, respectively. ω_{Fe} and $r_{Fe/S}$ increase gradually with increasing C_{FeCl_3} (0−0.3 M), indicating the gradually increased quantity of the formed metal-coordination bonds; they become comparable after $C_{FeCl_3} \geq 0.5$ M. As illustrated in **Figure S1**, Fe^{3+} ions can form three kinds of coordination patterns, i.e., mono-, bis-, and tris-complexes [61]. The strength of

the metal-coordination bond increases with increasing coordination number of Fe^{3+} ions. Metal-coordination bonds were formed between Fe^{3+} ions and $-SO_3^-$ groups in the networks. We hypothesize that there exists only one coordination pattern in the gels, and the molar ratio n(metal-ligand): n(ionic) ($r_{m/i}$) is calculated based on the atomic ratio of Fe:S (**Table S1**). With increasing C_{FeCl_3} (0−0.3 M), $r_{m/i}$ increases gradually, enabling gradually enhanced mechanical properties; After $C_{FeCl_3} \geq 0.5$ M, $r_{m/i}$ becomes relatively stable but the tensile properties of the gels decrease slightly, which is probably attributed to the mildly weakened quality of the formed metal-coordination bonds.

Table S2. Summary of tensile properties of WEQ-PNA gel, WEQ-PNA-Fe gel, WEQ-PA gel, and WEQ-PA-Fe gel shown in Figure S5. The original PA gel is P(NaSS-*co*-DMAEA-Q) gel, and the original PNA gel is P(NaSS-*co*-AAm) gel. C_M = 2.0 M, C_{FeCl_3}= 0.3 M.

Sample code	E (MPa)	ε_b (m m^{-1})	σ_b (MPa)	W_b (MJ m^{-3})
WEQ-PNA	0.007 ± 0.001	0.41 ± 0.02	0.003 ± 0.001	0.0007 ± 0.0002
WEQ-PNA-Fe	0.016 ± 0.001	4.3 ± 0.1	0.11 ± 0.02	0.13 ± 0.01
WEQ-PA	0.046 ± 0.002	5.1 ± 0.1	0.48 ± 0.01	0.68 ± 0.06
WEQ-PA-Fe	0.91 ± 0.09	6.1 ± 0.1	2.9 ± 0.06	5.6 ± 0.2

Table S3. Summary of tensile properties of neat WEQ-PA gels with different C_M (1.5–2.5 M).

Sample code (C_M) [a)]	σ_b (MPa)	E (MPa)	W_b (MJ m^{-3})
WEQ-PA-1.5	0.008 ± 0.002	0.006 ± 0.001	0.004 ± 0.001
WEQ-PA-1.7	0.27 ± 0.02	0.035 ± 0.002	0.39 ± 0.03
WEQ-PA-2.0	0.48 ± 0.01	0.05 ± 0.01	0.68 ± 0.06
WEQ-PA-2.3	0.79 ± 0.03	0.10 ± 0.02	1.4 ± 0.1
WEQ-PA-2.5	1.0 ± 0.1	0.18 ± 0.02	1.6 ± 0.2

[a)] C_M in the code of WEQ-PA-C_M represents the monomer concentration in pre-gel solutions.
